# Bone Microenvironment Specific Roles of ITAM Adapter Signaling during Bone Remodeling Induced by Acute Estrogen-Deficiency

**DOI:** 10.1371/journal.pone.0000586

**Published:** 2007-07-04

**Authors:** Yalei Wu, James Torchia, Wei Yao, Nancy E. Lane, Lewis L. Lanier, Mary C. Nakamura, Mary Beth Humphrey

**Affiliations:** 1 Department of Medicine, VA Medical Center, University of California San Francisco, San Francisco, California, United States of America; 2 Department of Microbiology and Immunology, University of California San Francisco, San Francisco, California, United States of America; 3 Department of Medicine, University of California Davis, Sacramento, California, United States of America; 4 Cancer Research Institute, University of California San Francisco, San Francisco, California, United States of America; 5 Department of Medicine and Microbiology and Immunology, VA Medical Center and University of Oklahoma Health Science Center, Oklahoma City, Oklahoma, United States of America; University of California, San Francisco, United States of America

## Abstract

Immunoreceptor tyrosine-based activation motif (ITAM) signaling mediated by DAP12 or Fcε receptor Iγ chain (FcRγ) have been shown to be critical for osteoclast differentiation and maturation under normal physiological conditions. Their function in pathological conditions is unknown. We studied the role of ITAM signaling during rapid bone remodeling induced by acute estrogen-deficiency in wild-type (WT), DAP12-deficient (*DAP12-/-*), FcRγ-deficient (*FcRγ-/-*) and double-deficient (*DAP12-/-FcRγ-/-*) mice. Six weeks after ovariectomy (OVX), *DAP12-/-FcRγ-/-* mice showed resistance to lumbar vertebral body (LVB) trabecular bone loss, while WT, *DAP12-/-* and *FcRγ-/-* mice had significant LVB bone loss. In contrast, all ITAM adapter-deficient mice responded to OVX with bone loss in both femur and tibia of approximately 40%, relative to basal bone volumes. Only WT mice developed significant cortical bone loss after OVX. *In vitro* studies showed microenvironmental changes induced by OVX are indispensable for enhanced osteoclast formation and function. Cytokine changes, including TGFβ and TNFα, were able to induce osteoclastogenesis independent of RANKL in BMMs from WT but not *DAP12-/-* and *DAP12-/-FcRγ-/-* mice. FSH stimulated RANKL-induced osteoclast differentiation from BMMs in WT, but not *DAP12-/-* and *DAP12-/-FcRγ-/-* mice. Our study demonstrates that although ITAM adapter signaling is critical for normal bone remodeling, estrogen-deficiency induces an ITAM adapter-independent bypass mechanism allowing for enhanced osteoclastogenesis and activation in specific bony microenvironments.

## Introduction

The osteoclast is the only bone-resorbing cell, and it develops from myeloid lineage cells, including macrophages and monocytes. It has been well established that receptor activator for nuclear factor κ B ligand (RANKL, also known as TRANCE, ODF, OPGL, and TNFSF11) and macrophage colony-stimulating factor (M-CSF) are required during osteoclast differentiation. Recent evidence suggests that immunoreceptor tyrosine-based activation motif (ITAM) containing adapter proteins, DAP12 or Fcε receptor I γ chain (FcRγ), are critical for osteoclast maturation and function under normal physiologic conditions *in vivo* and *in vitro* [1]–[4]. Upon activation, the tyrosines in the ITAM, YxxL/Ix6-8YxxL/I (where x represents any amino acid), are phosphorylated by members of the *Src* kinase family. The tyrosine kinase *Syk* is then recruited to initiate a signal transduction cascade, which includes phospholipase Cγ (PLCγ), calcium mobilization, phosphatidylinositol-3 kinase (PI3K), Ras, nuclear factor-κB (NFκB), and nuclear factor for activation of T cells (NFAT).

With minimal extracellular domains, DAP12 and FcRγ have no known ability to bind ligands. Therefore, they are insufficient to sense the local microenvironment alone. The DAP12 and FcRγ signaling adapters associate with innate immune transmembrane receptors that have ligand recognition domains. Innate immune receptors are genetically encoded to sense environmental changes and provide front line defense against infection. Certain activating innate immune receptors, such as Dectin-1 [5], use intrinsic ITAMs to transduce activating signals [1]; however, there is also a class of innate immune receptors that lack signaling motifs in their intracellular domains. Instead, these receptors with minimal cytoplasmic domains partner with ITAM-containing adapter proteins, like DAP12 or FcRγ, and form protein complexes to mediate their downstream signals [3], [6]. ITAM-bearing adapter-associated receptors expressed in preosteoclasts or osteoclasts include triggering receptor expressed on myeloid cells (TREM2), osteoclast-specific activating receptor (OSCAR), myeloid DAP12-associating lectin-1 (MDL-1), signal regulatory protein-β (SIRPβ), and paired-Ig-like receptor-α (PIRα) [1]. Depending on the structural properties of their transmembrane regions, these receptors will associate with either DAP12 or FcRγ to transduce downstream signals. Mice that are doubly deficient in both adapter proteins, DAP12 and FcRγ, have severe osteopetrosis with a trabecular bone volume/tissue volume (BV/TV) around 60% and single nucleated osteoclasts both *in vitro* and *in vivo* [4]. Mice that are deficient in a single adapter protein, either DAP12 or FcRγ, have moderate or mild osteopetrosis, respectively. These data indicate that ITAM signaling mediated by these distinct groups of receptor-adapter protein complexes can compensate partially for each other. Poorly functional osteoclasts in double-deficient mice suggest that ITAM-containing adapter-associated receptors and their ligands present in the microenvironment are critical for osteoclast differentiation in normal bone remodeling.

Specific ligands that activate ITAM signals in osteoclast precursors have not been identified. It is also unknown whether ligands in the bone microenvironment are subjected to change in different physiological conditions. The mouse ovariectomy (OVX) model has been a useful tool to study rapid bone remodeling, induced by estrogen-deficiency that proceeds after bilateral oophorectomy. During OVX-induced bone loss, several genes have been identified to be essential in osteoclastogenesis, including β3 integrin, MIF, IGF-I, thrombospondin-2 and PAI-1. The deficiency of these genes has been shown to protect mice from bone loss in OVX studies [7]–[11]; however, the role of ITAM signaling in bone remodeling during estrogen-deficiency has not been addressed.

Using the OVX model in mice that are deficient for either one or both ITAM-containing adapter proteins, we examined whether acute estrogen-deficiency would increase bone remodeling at several bone locations. In addition, we examined the role of ITAM signaling during osteoclast differentiation and functional changes after OVX *in vivo* and *in vitro.*

## Results

### Estrogen-deficiency induces bone remodeling in ITAM-adapter deficient mice

**Figure 1:**
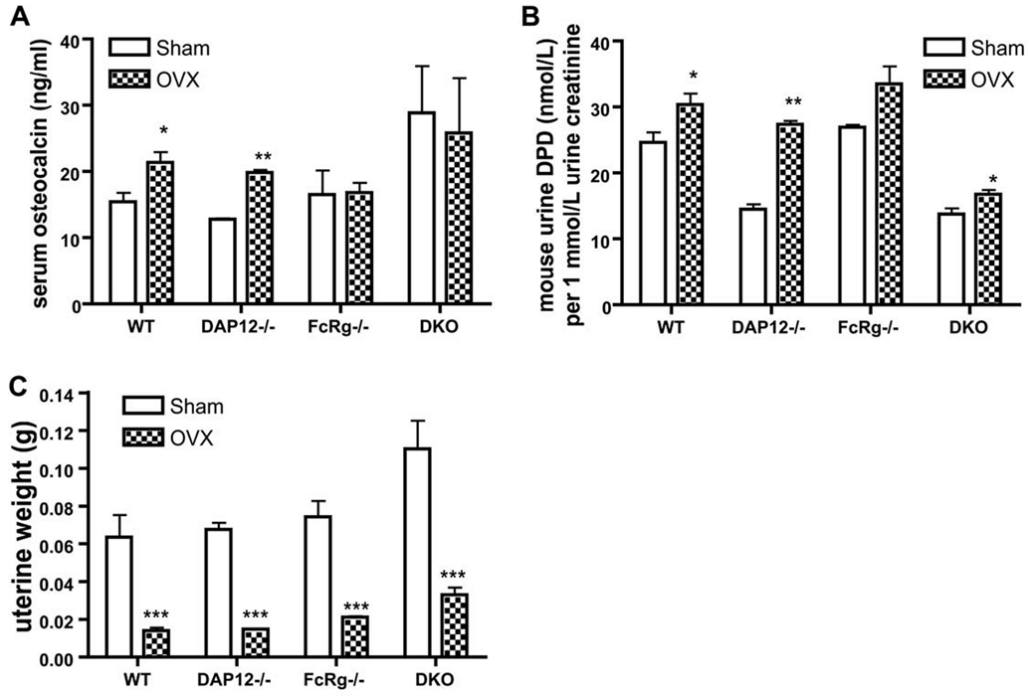
Bone remolding markers and uterine weights. (A) Uterine weight was measured at the end of the experiment when mice were sacrificed. Four weeks after operations, urine DPD (B) and serum osteocalcin (C) were measured. WT: wild-type mice; DKO: mice lacking both DAP12 and FcRγ. N = 5 in each group. *: p<0.05; **: p<0.01; ***: p<0.001; when compared to SHAM groups, respectively.

In this study, we used the ovariectomy model to study the physiological role of ITAM signaling in estrogen-deficiency-induced bone remodeling. Eight-week-old female mice underwent sham operation (SHAM) or bilateral ovariectomy (OVX), and bone remodeling was evaluated at 6 weeks post-procedure. At the time of sacrifice, uterine atrophy confirmed successful ovariectomy in all four OVX groups (Fig. 1A). At 4 weeks after operation, urine and blood samples were collected to evaluate bone remodeling, using serum osteocalcin, a bone formation marker, and urinary deoxypyridinoline (DPD), a bone resorption marker. Serum osteocalcin was increased in the OVX group of WT and *DAP12-/-* mice compared to their SHAM groups. Osteocalcin remained unchanged in the *FcRγ-/-* and *DAP12-/-FcRγ-/-* OVX mice (Fig. 1C). Urinary DPD was lower at basal level in both *DAP12-/-* and *DAP12-/-FcRγ-/-* SHAM mice compared to WT and *FcRγ-/-* SHAM groups, indicating abnormal osteoclast function at baseline (Fig. 1B). Urine DPD level was increased in all OVX groups independent of ITAM status, indicating increasing bone resorption.

### Estrogen-deficiency induces site-specific bone loss in ITAM adapter-deficient mice

**Figure 2:**
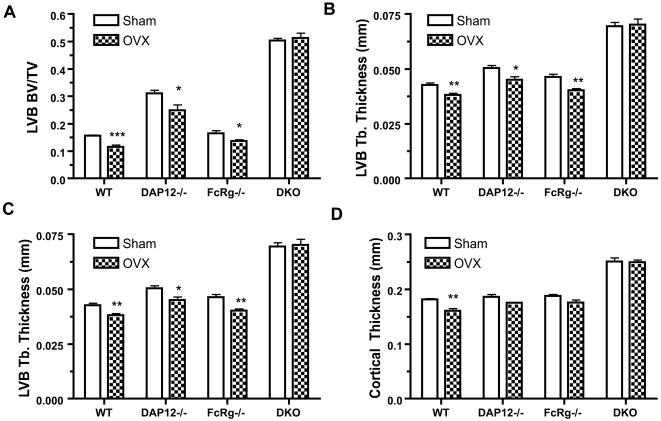
OVX-induced bone remodeling in lumbar vertebra and cortical bone. Six weeks after operations, the sixth lumbar vertebra (LVB6), and tibia were processed and analyzed by µCT, as described in Materials and Methods. Trabecular bone volume/tissue volume (A), trabecular thickness (B), and trabecular number (C) of LVB6, and cortical thickness of tibia at distal tibiofibular junction (D) are shown. WT: wild-type mice; DKO: mice lacking both DAP12 and FcRγ. N = 5 in each group. *: p<0.05; **: p<0.01; ***: p<0.001; when compared to SHAM groups, respectively.

MicroCT (µCT) of the trabecular region of lumbar vertebral body (LVB) at 6 weeks post-OVX was performed to determine the bone microarchitecture and bone mass (bone volume/ tissue volume; BV/TV). OVX induced significant bone loss in LVB of WT and *FcRγ-/-* mice. *DAP12-/-* mice had a baseline BV/TV of approximately 30% at the LVB, a doubling of bone mass compared to WT mice (p<0.001). Although *DAP12-/-* mice exhibited bone loss (5% of BV/TV) at this site, the bone mass remained significantly higher compared to SHAM WT (p<0.01). No significant bone loss occurred in the *DAP12-/-FcRγ-/-* OVX group (BV/TV = 50%) compared to SHAM (BV/TV = 51%) (Fig. 2A).

The microarchitectural analysis results of the µCT were consistent with the bone mass analysis. Both *DAP12-/-* and *DAP12-/-FcRγ-/-* SHAM groups had thicker trabeculae compared to WT and *FcRγ-/-* mice (Fig. 2B). 6 weeks post-OVX, WT, *DAP12-/-*, and *FcRγ-/-*mice had statistical significant decreases in the trabecular thickness (Fig. 2B). Notably, *DAP12-/-* and *DAP12-/-FcRγ-/-* mice had higher trabecular numbers compared to WT and *FcRγ-/-* mice (Fig. 2C). In all four groups of mice, only the *DAP12-/-* OVX group showed a statistically significant reduction in trabecular number. However, thinning of trabeculae and reduction in trabecular counts were not observed in the *DAP12-/-FcRγ-/-* OVX group compared to SHAM. These data suggest that ITAM adapter signaling is required for estrogen deficiency-related bone loss in the vertebrae, and the expression of either DAP12 or FcRγ ITAM adapter is sufficient to mediate bone remodeling signals in vertebrae after OVX.

Cortical bone remodeling has been demonstrated to respond to estrogen-deficiency in mice. Therefore, we determined whether ITAM-adapter signaling is required during this process. We analyzed cortical bone proximal to the tibiofibular joint by µCT. As shown in Fig. 2D, a statistically significant decrease in cortical thickness was only observed in WT OVX mice. *DAP12-/-* and *FcRγ-/-* mice showed a trend toward a decrease of trabecular thickness in OVX group, but didn't reach statistical significance at 6 weeks post-procedure. *DAP12-/-FcRγ-/-* mice had the thickest cortical bones and exhibited no change post-OVX. Thus, as in lumbar vertebrae, ITAM-adapter signaling is required for cortical bone loss induced by acute estrogen-deficiency.

### Estrogen-deficiency induced trabecular bone loss in long bones is ITAM-adapter independent

**Figure 3:**
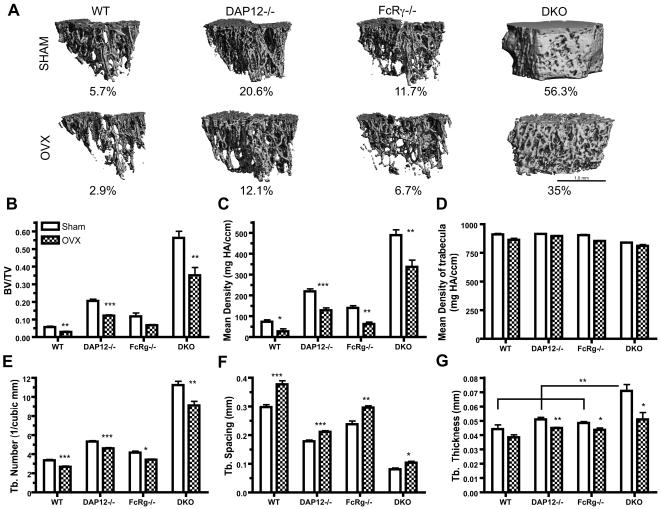
OVX-induced bone remodeling of trabeculae in distal femur. Six weeks after operations, femurs were processed and analyzed by µCT. Representative figures of 3D reconstructed µCT results are shown in (A), with average BV/TV indicated below. Trabecular BV/TV (B); mean bone mineral density of TV (C); mean bone mineral density of trabeculae (D); trabecular number (E); trabecular spacing (F) and trabecular thickness (G) are shown. Scale bar in (A) shows 1 mm physical size. WT: wild-type mice; DKO: mice lacking both DAP12 and FcRγ. N = 5 in each group. *: p<0.05; **: p<0.01; ***: p<0.001; when compared to SHAM groups, respectively. ** in (G) indicates p<0.01 when DKO sham group were compared to sham group of all three other mice. N = 5 for each group.

MicroCT evaluation of the distal femur and proximal tibia was performed at 6 weeks post-OVX or SHAM operations. Unlike the vertebrae and cortical bone, significant trabecular bone loss was observed in all ITAM-adapter deficient mice following OVX (Fig. 3A). The 3-D reconstructed µCT images of trabeculae in distal femur from both SHAM (Fig. 3A, upper panel) and OVX (Fig. 3A, lower panel) exhibited loss of trabecular bone in mice independent of ITAM-adapter status. Although all four mouse groups showed reductions in trabecular bone mass following OVX operation, *DAP12-/-FcRγ-/-* mice had the most significant change, as their trabecular bone changed from a more solid structure with few holes to a very porous sponge-like structure (Fig 3A).

BV/TV dropped significantly in WT from 5.7% to 2.9%, a 49% relative bone loss (Fig. 3B, Table 1). *DAP12-/-* mice showed a 41% relative trabecular bone loss (BV/TV dropping from 20.6% to 12.1%). A similar 38% relative bone loss was observed in *DAP12-/-FcRγ-/-* OVX group (BV/TV dropping from 56.2% to 35%) (Fig. 3A, Table 1). *FcRγ-/-* mice also showed similar relative bone loss of 43%, although the bone loss didn't reach a statistical significance (p = 0.09) (Fig. 3B, Table 1). The biggest decrease of BV/TV was seen in *DAP12-/-FcRγ-/-* mice, 21.3% compared to 2.8% in WT, 8.4% in *DAP12-/-* and 5% in *FcRγ-/-* mice.

**Table 1:** Bone loss in the trabecular region of the distal femur.

	WT	*DAP12-/-*	*FcRγ-/-*	DKO
BV/TV_SHAM_	5.7%	20.6%	11.7%	56.3%
BV/TV_OVX_	2.9%	12.1%	6.7%	35.0%
Δ	−2.8%	−8.4%	−5.0%	−21.3%
Δ/BV/TV_SHAM_ (x100%)	−49	−41	−43	−38

Measurements acquired at six weeks after OVX (N = 5).

Mean density of the trabecular region (Fig. 3C) showed strong correlation with BV/TV (Fig. 3B), whereas the mean density of trabeculae stayed constant in both groups from all four different mice (Fig. 3D), indicating the bone loss was due to structural destruction of trabeculae. µCT-based structural analysis showed similar findings. Consistent with dysfunctional osteoclasts under normal bone remodeling conditions, *DAP12-/-FcRγ-/-* mice had the highest trabecular number and thickness in the SHAM group, followed by *DAP12-/-* (Fig 3E, 3G). A statistically significant reduction in trabecular number was seen in all OVX groups at 6 weeks post operation compared to their SHAM groups (Fig. 3E). Smaller trabecular spacing was also observed in all ITAM-adapter deficient mice SHAM groups compared to WT SHAM (Fig. 3F). Among them, *DAP12-/-FcRγ-/-* mice had the smallest spacing, about 1/3 of WT (Fig. 3F). OVX induced a statistically significant increase in trabecular spacing in all four OVX groups, including *DAP12-/-FcRγ-/-* mice (Fig. 3F). OVX induced a decrease in trabecular thickness in all four OVX groups compared to the SHAM groups (Fig. 3G). Interestingly, while *DAP12-/-FcRγ-/-* OVX group still maintained higher trabecular number and smaller trabecular spacing among all OVX groups (p<0.05), the difference in trabecular thickness was no longer statistically significant after OVX (Fig. 3G). Similar bone losses were observed in the proximal tibia at 6 weeks in all OVX groups (data not shown). Thus, ITAM-adapter signaling is not required for estrogen-deficient bone loss in the trabecular rich regions of long bones, indicating that these bone microenvironments have an ITAM-adapter independent mechanism to generate active osteoclasts. Furthermore, mice deficient in DAP12 alone or in combination with FcRγ deficiency had significantly more absolute bone loss than WT mice, suggesting loss of an inhibitory signal required to limit bone loss induced by OVX.

### Estrogen-deficiency induces an increased number of multinucleated osteoclasts in DAP12-/-FcRγ-/- mice in vivo

Although *DAP12-/-FcRγ-/-* mice failed to lose trabecular bone in their vertebrae and cortical areas under estrogen-deficient conditions, they showed a substantial trabeculae bone loss of 38% in long bones. *DAP12-/-FcRγ-/-* osteoclasts have been shown to be defective in function both *in vivo* and *in vitro,* with mononuclear osteoclasts having poor bone resorbing ability [3], [4]. To determine the mechanism allowing significantly increased bone remodeling post-OVX in *DAP12-/-FcRγ-/-* mice, we looked for *in vivo* evidence of OVX-induced osteoclast generation or function.

**Figure 4:**
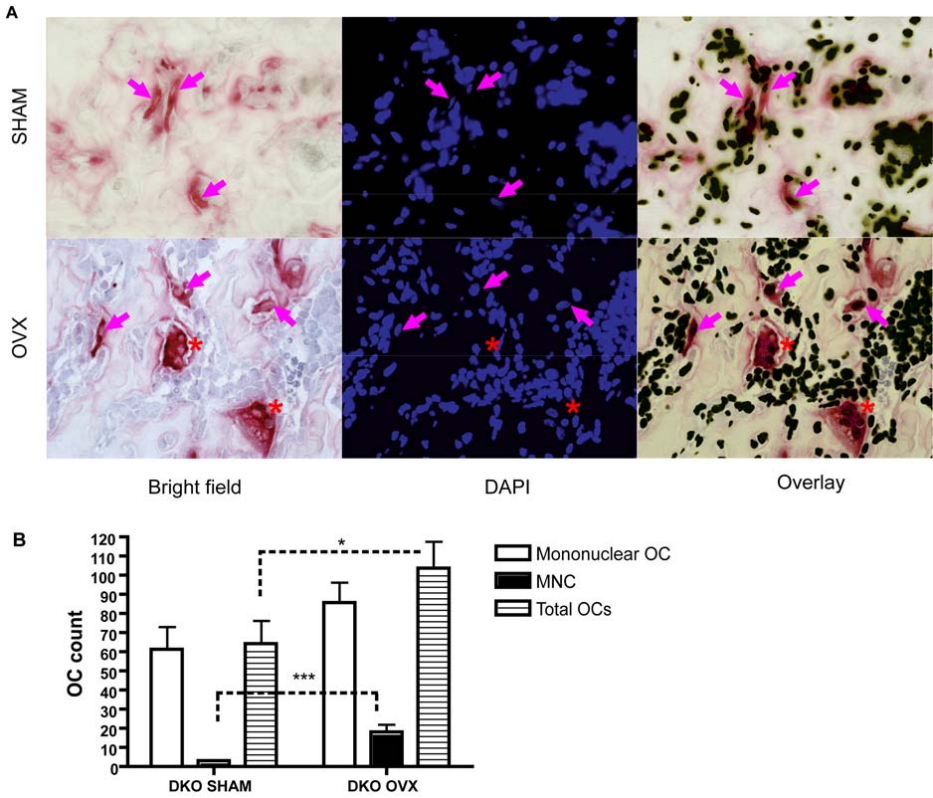
Increased osteoclast formation *in vivo* in *DAP12-/-FcRγ-/-* OVX group. (A) TRACP staining and DAPI staining was performed on paraffin section of decalcified femur of SHAM and OVX groups of DKO mice. Brightfield and fluorescence images were taken with 40× objective. Red staining in brightfield indicates TRACP^+^cells. Magenta arrow indicates mononuclear TRACP^+^ osteoclasts; red asterisk indicates TRACP^+^ multinuclear osteoclasts (MNCs). (B) Mononuclear, multinuclear, and total osteoclast number per section was counted. *: p<0.05; ***: p<0.001; when compared between groups indicated by dotted line. N = 3 each group.

6 weeks post-operations, femurs from *DAP12-/-FcRγ-/-* mice were processed for histological examination. Consistent with the µCT analysis, the bone marrow cavities were small in the SHAM group (Fig. 4A, upper panel), compared to the OVX group (Fig. 4A, lower panel). In the *DAP12-/-FcRγ-/-* SHAM group, mononucleated TRACP-positive osteoclasts were identified (indicated by purple arrows in Fig. 4A, upper panel), while only a few multinucleated osteoclasts were identified (data not shown). In the OVX group, a significant increase in multinucleated osteoclast number was observed (p = 0.0008) (indicated by red asterisk in Fig. 4A, lower panel, Fig. 4B). The multinucleated osteoclasts had increased size and revealed stronger TRACP staining. The mononucleated osteoclast number was also increased in the *DAP12-/-FcRγ-/-* OVX group, but the difference was not statistically significant (p = 0.14) (Fig. 4B). Total osteoclast number was significantly higher in OVX compared to SHAM (Fig. 4B).

Histomorphometry study on lumbar vertebrae from WT, *DAP12-/-*, and *FcRγ-/-* mice is consistent with our µCT analysis. Osteoclast surface and osteoclast surface per bone surface were increased in *DAP12-/-* and *FcRγ-/-* OVX mice, reaching statistical significance in both parameters only in *FcRγ-/-* mice (p<0.05). Osteoclast number increased 50% in *DAP12-/-* OVX (p = 0.28) and 80% in *FcRγ-/-* OVX (p = 0.063) groups. Mineralizing surface was decreased 25% in *DAP12-/-* OVX mice (p = 0.07) and was increased 51% in *FcRγ-/-* OVX mice (p = 0.15), indicating a differential bone formation response in ITAM-adapter deficient mice. These data are consistent with an ITAM-adapter independent increase in osteoclast number and activity as the main mechanism behind the acute estrogen-deficient bone loss observed in ITAM-adapter deficient mice.

### Increased osteoclast function induced by OVX is not sustained in vitro from DAP12-/- and DAP12-/-FcRγ-/- mice

**Figure 5:**
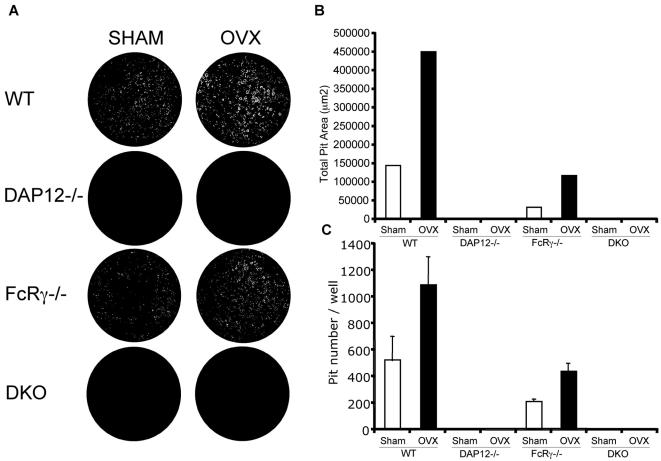
OVX-induced osteoclast activity is not self-sustained in osteoclasts from *DAP12-/-* and *DAP12-/-FcRγ-/-* mice. At six weeks post-OVX, non-adherent bone marrow macrophages were cultured with 10 ng/mL M-CSF and 25 ng/mL RANKL on BD BioCoat™ Osteologic™ Bone Cell Culture dishes. (A) Representative images of resorption pits where white represents pits and black in unresorbed substrate. Total resorption area (B) and pit number (C) were calculated and shown. Standard error is shown. WT: wild-type mice; DKO: mice lacking both DAP12 and FcRγ. N = 5 in each group. Experiment was repeated three times. Representative images and results were shown.

An increased number of multinucleated osteoclasts *in vivo* in *DAP12-/-FcRγ-/-* mice post-OVX indicates that the requirement for ITAM-adapter costimulation may be bypassed during estrogen-deficiency. To investigate if this compensatory mechanism is intrinsic to the osteoclast progenitor lineage, we analyzed *in vitro* osteoclast differentiation and function post-OVX. In order to recapitulate the increased osteoclast function *in vitro*, we cultured preosteoclasts from bone marrow-derived macrophages (BMM) from both OVX and SHAM groups, seeded them on an artificial calcium-phosphate substrate (BD BioCoat), and measured resorption. Preosteoclasts were cultured with M-CSF and RANKL for 7 days. Resorption areas were visualized with microscopy and digital images captured (Fig. 5A). As expected, *in vitro* cultured osteoclasts from the WT OVX group had increased number and size of resorption pits and the total resorption area was significantly increased (about 2-fold) compared to the WT SHAM group (Fig. 5B, 5C). Similar to WT, osteoclast cultures from *FcRγ-/-* OVX showed a significant increase in resorption pit number and total resorption area compared to *FcRγ-/-* SHAM (Fig. 5B, 5C). Interestingly unlike WT and *FcRγ-/-* mice, osteoclast cultures from *DAP12-/-* and *DAP12-/-FcRγ-/-* mice did not show an increase in resorption area in the resorption assay (Fig. 5A, 5B, 5C). Therefore, the osteoclast cultures from WT and *FcRγ-/-* mice could sustain the stimulatory signaling of estrogen-deficiency in the *in vitro* culture; whereas *DAP12-/-* and *DAP12-/-FcRγ-/- in vitro* derived osteoclasts had no increase in *in vitro* resorption capacity post-OVX. Co-culture of stromal cells and BMMs from *DAP12-/-* and *DAP12-/-FcRγ-/-* OVX groups failed to rescue enhanced osteoclast generation or function (data not shown). These results indicate that the increase in osteoclast number and size *in vivo* is not due to an osteoclast intrinsic change but to a microenvironmental change permissive to ITAM-adapter independent osteoclastogenesis.

### TNFα stimulation fails to rescue ITAM-adapter deficient osteoclast defects

**Figure 6:**
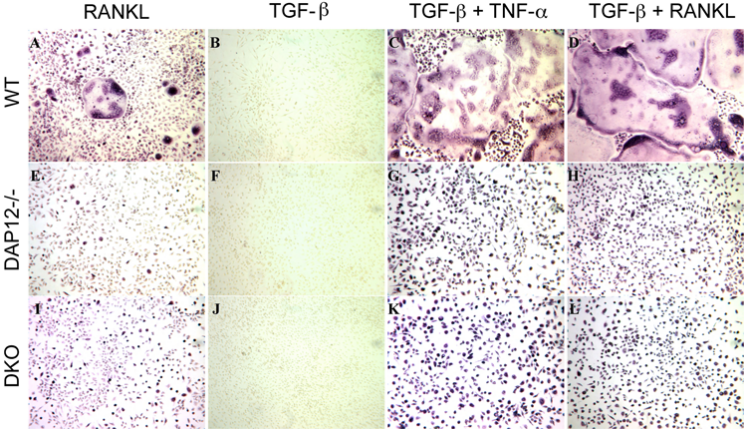
RANKL-independent osteoclast differentiation induced by TGFβ and TNF-α cytokines. BMMs from WT, *DAP12-/-* and *DAP12-/-FcRγ-/-* were separated and non-adherent preosteoclasts were cultured with RANKL (A, E, I); or pretreated by 2 ng/ml TGFβ (B, F, J) for 2 days, followed by 30 ng/ml TNF-α treatment (C, G, K) or 25 ng/ml RANKL (D, H, L) for another 2 day. TRACP staining was performed at the end of the experiment, as described. TRACP staining is indicated by dark purple. WT: wild-type mice; DKO: mice lacking both DAP12 and FcRγ. Experiment was repeated three times with representative results shown.

The essential role of RANKL during osteoclastogenesis was challenged by Kim *et al.* [12] when they demonstrated that osteoclast differentiation *in vitro* could be induced by TNF-α independent of the RANK-TRAF6 axis. Their data suggested alternative routes for osteoclast differentiation. It's known that estrogen-deficiency can induce systemic cytokine changes (such as TNFα, IL6, IL1, IL7, and M-CSF) [13], [14] and in the bone resorbing lacunae. In order to determine a possible mechanism for ITAM-adapter independent osteoclastogenesis induced by OVX, we examined the effect of cytokine stimulation on osteoclast differentiation with ITAM-adapter signaling deficiency. BMM from WT, *DAP12-/-*, and *DAP12-/-FcRγ-/-* mice were treated with TGFβ for two days, followed by TNFα or RANKL stimulation. As shown in Fig. 6, TGFβ treatment alone failed to induce any TRACP activity in all groups, as indicated by the lack of TRACP^+^-staining cells (Fig. 6B, 6F, 6J). TGFβ treatment also potentiated the effect of RANKL in wild-type osteoclast cultures, forming large, more complex multinucleated cells (Fig. 6D, 6H, 6L) compared to RANKL treatment alone (Fig. 6A, 6E, 6I). Additionally, in the absence of RANKL, TNFα following TGFβ treatment effectively induced multinucleated TRACP^+^ osteoclast formation in WT BMM. RANKL-treated BMMs from *DAP12-/-* or *DAP12-/-FcRγ-/-* mice formed mononucleated TRACP^+^ cells and failed to form multinucleated cells (Fig. 6C, 6G, 6K), consistent with previous studies showing that high dose RANKL could not rescue the multinucleation defect in *DAP12-/-* cells [4]. TGFβ pre-treatment increased the formation of TRACP^+^ mononuclear osteoclasts in *DAP12-/-* and *DAP12-/-FcRγ-/-* cultures approximately 2-fold compared to RANKL or TNFα (Fig. 6G, 6H, 6K and 6L). However, TGFβ pre-treatment failed to rescue the osteoclast multinucleation defect in *DAP12-/-* and *DAP12-/-FcRγ-/- in vitro* cultures (Fig. 6H, 6L). Thus, microenvironmental cytokine changes induced by estrogen-deficiency including TGFβ and TNFα were unable to permit ITAM-adapter independent osteoclastogenesis.

### FSH fails to rescue the ITAM deficient osteoclast defect

**Figure 7:**
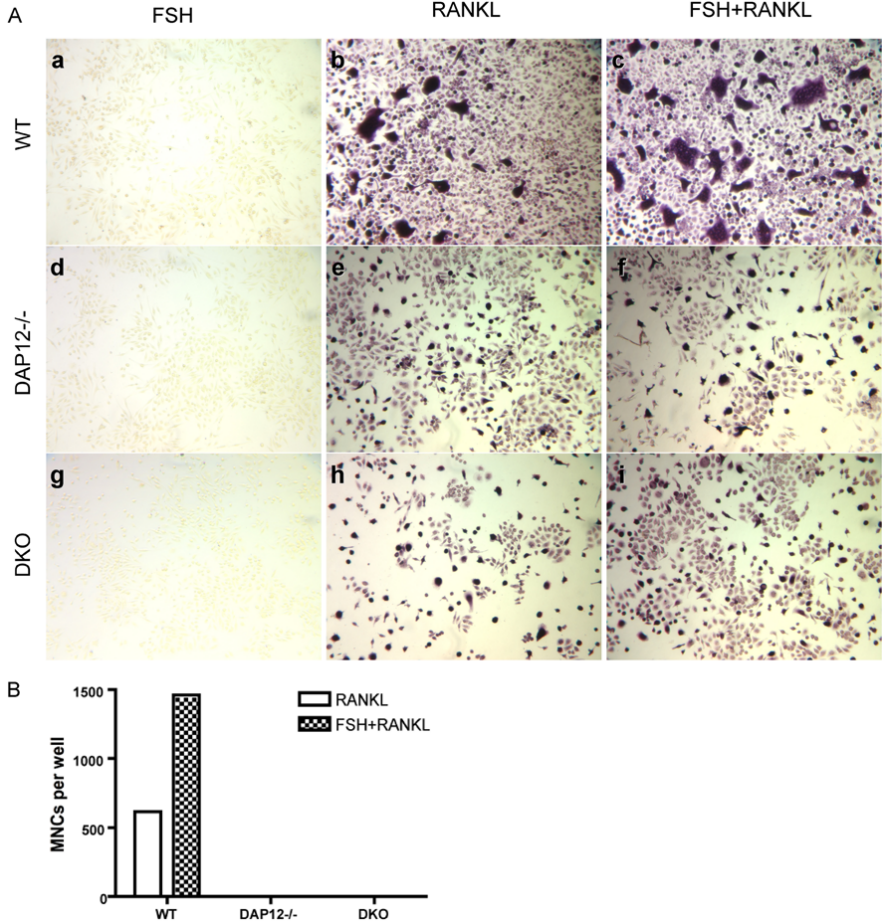
Stimulatory effect of FSH has no effect on DAP12-deficient osteoclastogenesis. BMMs from WT, *DAP12-/-*, and *DAP12-/-FcRγ-/-* were separated and non-adherent BMMs were cultured with 30 ng/ml FSH alone (Aa, Ad, Ag), 3 ng/ml RANKL alone (Ab, Ae, Ah), or FSH and RANKL (Ac, Af, Ai) for 4 days. TRACP staining was performed at the end of the experiment, as described. Dark purple staining indicates that cells are TRACP positive. The number of multinucleated (>3nuclei) -osteoclasts were calculated and shown in (B). DKO: mice lacking of both DAP12 and FcRγ. Experiment was repeated three times with representative results shown.

Recent reports suggest that increased FSH levels induced during estrogen-deficiency play an important role in stimulating osteoclast formation during postmenopausal osteoporosis [15]. FSH was shown to stimulate RANKL induced osteoclastogenesis, including increased multinucleation. To determine whether FSH could potentiate osteoclast differentiation in ITAM-adapter deficient mice, we evaluated the effect of FSH during *in vitro* osteoclastogenesis. BMM from WT, *DAP12-/-*, and *DAP12-/-FcRγ-/-* were cultured with 30 ng/ml FSH and 3 ng/ml RANKL for 3 days followed by TRACP staining. FSH treatment alone failed to induce any TRACP activity in BMM cultures independent of ITAM-adapter status (Fig. 7Aa, Ad, Ag). Compared to RANKL treatment alone, FSH and RANKL co-treatment increased TRACP^+^ multinucleated osteoclast formation 3-fold in WT cultures (Fig. 7Ac, 7B). Unlike WT cultures treated with FSH and RANKL, there was no increase in the number of mononuclear or multinuclear TRACP^+^ osteoclasts in *DAP12-/-* and *DAP12-/-FcRγ-/-* cultures. Additionally, FSH treatment alone or in combination with RANKL failed to rescue the *in vitro* osteoclast fusion defect in *DAP12-/-* and *DAP12-/-FcRγ-/-* mice (Fig. 7Ae, f, h, I; 7B). Whereas FSH synergistically increased WT osteoclastogenesis, we conclude that FSH was unable to drive ITAM-adapter independent osteoclastogenesis.

## Discussion

ITAM-mediated signaling has been shown to be critical for osteoclast differentiation and function under normal physiological conditions. ITAM signaling requires multiple components including ITAM-containing adapter proteins, adapter-associated downstream signal mediators, receptors, and their putative ligands. Variation in the local expression any of these ITAM signaling components may contribute to the changes in bone remodeling observed in unique bone microenvironments. The deficiency of ITAM-containing adapter proteins leads to a disconnection of intracellular signaling from the osteoclast microenvironment sensed by the receptors mediating these ITAM signals. Here we show that acute estrogen-deficiency induced osteoporosis is differentially affected by deficiency of ITAM adapter proteins. ITAM-adapter deficiency (*DAP12-/-FcRγ-/-*) leads to a complete blockade OVX induced bone loss in vertebrae and cortical bones. However, quite unexpectedly, ITAM signaling is bypassed in the estrogen-deficient state with losses of significant amounts of trabecular bone in the femur and tibia. More interesting, the mechanism bypassing ITAM signaling was not readily explained by our current understanding of requirements for osteoclastogenesis.

Severe osteopetrosis in the *DAP12-/-FcRγ-/-* mice suggests that ITAM-adapter signaling plays an important role in normal bone remodeling. Unlike other severely osteopetrotic mice, such as those deficient in MCSF-1 [16], PTHrP [17], c-fos [18], or RANKL [19], *DAP12-/-FcRγ-/-* mice have tooth eruption, suggesting that there are mechanisms that allow normal osteoclastogenesis in the absence of ITAM-adapter signals. Thus, even under normal physiological conditions, distinct localized regulation of bone remodeling in *DAP12-/-FcRγ-/-* mice already exists. Signals that can bypass the need for ITAM-adapter signaling to induce the necessary osteoclast differentiation activity for tooth eruption are not known. Severe osteopetrosis in the long bones in *DAP12-/-FcRγ-/-* mice suggests that during normal physiological conditions osteoclastogenesis is largely controlled by ITAM-adapter signals transduced through a ligand-receptor-adapter axis, presumably involving interactions with the microenvironment. The exception of tooth eruption and bone mobilization during estrogen-deficiency suggests that there is an alternate signaling pathway that can be elicited in specific sites during certain stresses to induce bone remodeling.

Here we showed that during bone remodeling following acute estrogen-deficiency, the defect of osteoclastogenesis and function could be rescued in ITAM-adapter deficient mice in trabecular long bone. Our data suggest that during a physiological state of estrogen-deficiency, such as lactation, *DAP12-/-FcRγ-/-* mice would be able to mobilize calcium to nutritionally support bone growth of the next generation. This emergency signaling pathway, which is quiescent during normal physiological conditions, activates osteoclasts and mobilizes mineral without utilizing ITAM-adapter signaling. We applied different models and theories to expand our understanding about how ITAM-adapter signaling is bypassed during estrogen-deficiency in ITAM-adapter deficient mice. Estrogen-deficiency can rescue the osteoclast defect of ITAM-adapter deficient mice stimulating osteoclast multinucleation and promoting bone resorption *in vivo*. However, unlike wild-type mice, this enhanced osteoclastogenesis was not intrinsic to BMMs or preosteoclasts in ITAM-adapter deficient mice, as shown in the *in vitro* cultures. Secondly, estrogen-deficiency induces changes in cytokine and hormone levels, including increased levels of TNFα and FSH. Both TNFα and FSH have been shown to stimulate or even substitute for RANKL during osteoclastogenesis through different mechanisms. TNFα, in combination with TGFβ, can induce osteoclast differentiation and multinucleation in the absence of RANKL in normal osteoclasts derived *in vitro* [12]. Increasing levels of FSH can cooperate with RANKL to have a synergistic effect on osteoclast differentiation during estrogen-deficiency *in vitro* [15]. However, as shown in this study, these two new models could not explain the increase in multinucleated osteoclasts seen in the *DAP12-/-FcRγ-/-* OVX mice.

Although we investigated alternative methods of osteoclast activation, one important mechanism that we did not address is the role of T lymphocytes in acute estrogen-deficiency induced bone loss. However, Pierre Jurdic and colleagues (Lyons, France) have recently investigated the role of mature lymphocytes and ITAM-adapter signaling in bone remodeling induced by OVX (A. Anginot, *et. al.*, PLoS One, in press). They have completed similar studies examining the role of DAP12 signaling and mature T and B cells in the acute estrogen deficient state in constitutively inactive DAP12 (*KΔ75*), RAG1 (*RAG1-/-*), or double deficient (*RAG1-/-*; *KΔ75*) mice. Their results demonstrate increased bone loss in the double deficient (*RAG1-/-; KΔ75*) mice suggesting that DAP12 ITAM signals negatively regulate bone remodeling in *RAG1-/-* mice consistent with our observations. Furthermore, *RAG1-/-* and *RAG1-/-;KΔ75* mice lose significant bone after OVX indicating that mature T and B cells are not needed for estrogen-deficiency induced bone loss and do not provide the missing signal for osteoclastogenesis. Combined with our own studies, these results strongly suggest a novel, still to be discovered, osteoclast activation mechanism *in vivo*.

The complexity of the different roles of ITAM-adapter signaling in different local microenvironments is consistent with the nature of the associated receptors. TREM2 and OSCAR are immunoglobulin superfamily innate immune receptors expressed on osteoclasts. TREM2, associated with DAP12, is considered a putative pattern-recognition receptor (PRR) because it recognizes a wide variety of anionic ligands, including dextran sulfate and bacterial products such as lipoteichoic acid and peptidoglycan [20]. PRRs are genetically coded and able to recognize conserved structural features in a large range of pathogens. Being able to sense these environmental changes, PRRs could respond upon ligand binding as a first line of defense. Presently, specific ligands in the bone for these two receptors are still unidentified.

Recent findings convincingly show that ITAM-containing adapter-receptor protein complexes can also send inhibitory signals, although the mechanism is still unclear [21], [22]. Despite the general concept that the ITAM in the intracellular domain of DAP12 will transduce an activation signal upon ligand binding of the associated receptor, it has been shown by multiple groups that DAP12-deficient macrophages have an enhanced response to TLR stimulation [22]. It has also been shown that TREM2, but not other DAP12-associated receptors, is responsible for transducing the inhibitory signal [23] [24]. It has been suggested that the type of downstream signals induced (activating or inhibitory) is dependent on the affinity and avidity of the extracellular ligands. High affinity or avidity ligands have been proposed to initiate activation, whereas low affinity or avidity ligands may cause inhibition [21].

We have observed evidence of a dual function of ITAM-adapter signaling in osteoclasts. The absence of downstream activation signal in ITAM-adapter deficient mice accounts for the osteopetrosis and defective osteoclastogenesis in ITAM-adapter deficient mice during normal physiological conditions. In our study, *DAP12-/-FcRγ-/-* mice lose about 40% trabecular long bone following OVX, which is similar to wild type, *DAP12-/-*, and *FcRγ-/-* mice. However *DAP12-/-FcRγ-/-* and *DAP12-/-* mice have much higher baseline bone mass. If the absolute bone loss is taken into consideration, *DAP12-/-FcRγ-/-* mice and *DAP12-/-* quantitatively lose more bone compared to wild-type mice. One possible explanation is that a low affinity ligand in the bone marrow compartment is engaged by TREM2/DAP12 that initiates an inhibitory signal in osteoclasts through DAP12 to fine-tune the resorption magnitude. In the absence of both ITAM-adapter proteins, the “inhibitory” signals cannot be used to regulate bone resorption, which leads to an extremely high absolute trabecular bone loss in *DAP12-/-FcRγ-/-* and *DAP12-/-* OVX mice. Thus, ITAM signaling adapters may have multiple functions in the regulation of osteoclasts. It will be necessary to identify these putative ligands in the bone to gain further insight into signals regulating these responses to local microenvironmental changes at distinct location and different stimuli in bone remodeling.

## Materials and Methods

### Mice

All animal procedures were performed in accordance with American Association for the Accreditation of laboratory Animals (AAALC) guidelines and were approved by the VA Animal Care Committee. Mice were maintained under sterile conditions. WT, *DAP12-/-, FcRγ-/-* and *DAP12-/-FcRγ-/-* mice were on a C57BL/6 background. C57BL/6 and *FcRγ-/-* was purchased from Jackson Laboratory (Bar Harbor, ME). *DAP12-/-* mice were kindly provided by Dr. Lewis L. Lanier (University of California at San Francisco). *DAP12-/-FcRγ-/-* mice were kindly provided Dr. Cliff Lowell (University of California at San Francisco). Female mice around 8–12 weeks of age were either subjected to sham or bilateral ovariectomy operation. Blood and urine sample was collected at 4 weeks post operation. At 6 weeks post operation, mice were sacrificed. Femur, tibia and lumbar vertebrate were excised for histology and micro-computed tomography analysis. Bone marrow was harvested for *in vitro* culture analysis. Uterine weight was determined to verify uterine atrophy.

### Bone turnover marker measurement

Blood and urine samples were collected 4 weeks post operation. Serum osteocalcin, a bone formation marker, was measured by mouse osteocalcin ELISA kit (Biomedical Technologies, Inc., Stoughton, MA) according to manufacture's protocol. Urine deoxypyridinoline, a bone resorption marker, was measured by METRA^®^ DPD (Quidel Corp., San Diego, CA) according to manufacture's protocol. Amount of urine DPD was standardized with creatinine using METRA^®^ creatinine kit (Quidel Corp., San Diego, CA).

### Micro-Computed Tomography (CT) Analysis

As previously described [4], [25], proximal tibias and distal femurs were scanned at the end of the experiment. Briefly, after being fixed serially in 4% formaldehyde and 70% ethanol, the proximal tibias and distal femurs were scanned by high resolution Scanco vivaCT 40 µCT scanner (Scanco Medical, Bassersdorf, Switzerland) with a resolution size of 10.5 µm. For proximal tibia and distal femur, scanning was initiated proximal to growth plate, and a total of 120 consecutive 10.5-µm-thick sections were analyzed. Cortical bone was excluded from the region of interest. The segmentation values were set constantly at 0.7/1/250. Cortical bone analysis was done at the tibia proximal to tibiofibular junction with a total of 60 consecutive 21-µm-thick sections. Stromal cavity was excluded from the region of interest with 30 slices evaluated. The segmentation values were set at 0.7/1/250. Three-dimensional reconstruction and structural parameters quantification were calculated using Scanco Medical software.

### BMMs culture and osteoclastogenesis

Bone marrow monocyte/macrophage (BMM) precursor cells were flushed out from femurs and tibias by 25-G needle. Cells were washed by PBS and lysed with RBC lysis buffer (0.16 M NH_4_Cl, 0.17 M Tris; pH 7.65) for 5 minutes at room temperature, and washed by PBS. Cells were cultured in complete α-MEM (Invitrogen, Carlsbad, CA, USA) supplemented with 10% fetal bovine serum, 1% glutamine Pen-Strep, and 10–20 ng/ml M-CSF (Sigma-Aldrich, St. Louis, MO). After 2 days in culture with M-CSF, non-adherent BMMs were transferred to a new plate at a density of 100,000 cells/well in 96-well plate or 250,000 cells/well in 24-well plate. BMMs were cultured in complete α-MEM with 25 ng/ml RANKL (PeproTech, Rocky Hill, NJ) with 10 ng/ml M-CSF for an additional 3–5 days, with fresh media every 3 days.

### TRACP staining

After 3–5 days in culture, cells were fixed in 4% formaldehyde in PBS for 10 minutes. Plates were washed with PBS, incubated with 0.05% Triton X-100 in PBS for 10 minutes at room temperature. Cells were stained for TRACP using a commercial kit (product 387-A; Sigma-Aldrich, St. Louis, MO) according to manufacture's protocol. Multinucleated (>2 nuclei) TRACP^+^ cells were counted and scored under microscopy.

### Histology staining

Six weeks post-operation, femurs were dissected and fixed with 4% formaldehyde for 12 hours at room temperature. Femurs were decalcified at 4°C for 1 week by decalcification buffer (10% EDTA [w/w] in 1× PBS), which was changed every other day. The bone samples were then processed and mounted in paraffin and sectioned into 5 µm thickness. TRACP staining was performed as described above. Methyl Green and DAPI (Invitrogen, Carlsbad, CA) were used for nuclear counterstaining for bright-field and fluorescence imaging. To create the overlay image, the DAPI fluorescent image was pasted on top of the bright-field image as a layer in Adobe Photoshop CS2 (Adobe Systems, Inc., San Jose, CA). By choosing “Difference” as the “Blend Mode” option, two layers were flattened to yield the overlay image.

### BD BioCoat assay

Bone marrow macrophages were harvested as described above. Macrophages were plated on BD BioCoat Osteologic Discs (BD Biosciences) at density of 100,000 cells/well and were treated with RANKL 25 ng/ml and M-CSF 10 ng/ml for 5–10 days. Media were changed every 3 days. BD BioCoat discs were treated with 20% bleach and agitated for 5 minutes to remove adherent cells, followed by 5 washes with dH_2_O and air-dried. The surface area with active bone resorption was visualized under Olympus microscope BX51 and measured by using BioQuant OSTEO II software (BioQuant Image Analysis Corporation, Nashville, TN).
